# Paeoniflorin Potentiates the Inhibitory Effects of Erlotinib in Pancreatic Cancer Cell Lines by Reducing ErbB3 Phosphorylation

**DOI:** 10.1038/srep32809

**Published:** 2016-09-09

**Authors:** Jian Hao, Xue Yang, Xiu-li Ding, Lei-ming Guo, Cui-hong Zhu, Wei Ji, Tong Zhou, Xiong-zhi Wu

**Affiliations:** 1 Zhong-Shan-Men Inpatient Department; National Clinical Research Center for Cancer; Key Laboratory of Cancer Prevention and Therapy; Tianjin Medical University Cancer Institute and Hospital Tianjin, 300060, China; 2 Clinical Immunology and Rheumatology, Medicine Department of University of Alabama at Birmingham, Birmingham, Alabama, USA; 3 Opening Cancer Laboratory, Tianjin Medical University Cancer Institute and Hospital, Tianjin 300060, China

## Abstract

Blockade of the epidermal growth factor receptor (EGFR) by EGFR tyrosine kinase inhibitors is insufficient for effective anti-tumor activity because the reactivation of the ErbB3 signaling pathway significantly contributes to activating the consequent phosphoinositide 3-kinase (PI3K)/Akt signaling pathway. Combinatorial therapies including ErbB3 targeting may ameliorate tumor responses to anti-EGFR therapies. In the present study, we found that in BxPC-3 and L3.6pl cells, which highly expressed the ErbB3 receptor, significant reduction in cell viability, induction of apoptosis were observed when treated with a combination of erlotinib and PF compared to either agent alone. Moreover, in ErbB3-expressing BxPC-3, L3.6pl and S_2_VP_10_ cell lines, the inhibition of ErbB3/PI3K/Akt phosphorylation were observed when treated with PF. Most strikingly, both EGFR/MAPK/Erk and ErbB3/PI3K/Akt activitions were substantially suppressed when treated with the combination of PF and erlotinib. However, in the ErbB3-deficient cell line MIAPaCa-2, no such effects were observed with similar treatments. Most importantly, these *in vitro* results were replicated in nude mouse transplanted tumor models. Taken together, our findings show that PF enhances the effect of erlotinib in ErbB3-expressing pancreatic cancer cells by directly suppressing ErbB3 activation, and PF in combination with erlotinib is much more effective as an antitumor agent compared with either agent alone.

Pancreatic cancer is a devastating disease with a five-year survival rate of <10%. The incidence of pancreatic cancer nearly equals its death rate (95.8%)^1^. Surgery remains the most effective treatment for pancreatic adenocarcinoma. However, only approximately 10–20% of cases are suitable for tumor resection, and fewer than 3% of patients are cured of this disease^2^. Unfortunately, pancreatic cancer has an unusual resistance to both radiation and chemotherapy. Only 5–10% of patients with metastatic pancreatic adenocarcinoma respond to chemotherapy, with a median survival of 5.7–6.8 months^3^,^4^.

EGFR is over-expressed in up to 60% of human pancreatic adenocarcinomas^5^. Erlotinib, orally bioavailable inhibitor of EGFR, in combination with gemcitabine has been recently clinically approved for nonresectable pancreatic cancer^6^. However, the benefit of EGFR blockade in pancreatic adenocarcinomas is small for the EGFR independent activation of ErbB3/PI3K/Akt^7^. ErbB3, a member of the EGFR family, plays a pivotal role in pancreatic tumourigenesis through heterodimerization with the other family members. The most intriguing feature of ErbB3 is that it contains multiple binding sites for the p85 regulatory subunit of PI3K, allowing it to activate the Akt pathway^8^,^9^,^10^,^11^. It has been previously reported that sensitivity to EGFR family TKI therapy correlates with the inhibition of ErbB3/PI3K/Akt pathway signaling due to the following reasons^12^,^13^,^14^,^15^,^16^,^17^,^18^. First, erlotinib affects sustained inhibition of EGFR phosphorylation and durable inhibition of downstream MAPK and JNK pathway signaling, but merely transient phosphorylation of the kinase-inactive family member ErbB3 through inhibiting its trans-phorsphorylation via EGFR^13^,^14^. Furthermore, other tyrosine kinases restore the ErbB3/PI3K/Akt signaling pathway and reduce the effect of EGFR targeting therapy, such as the amplification of c-MET^19^, over-expression of heregulin/ErbB3^20^, and high expression of IGF-1R^21^,^22^. The recovery of ErbB3 phosphorylation leads to pancreatic cancer cell lines, which are initially sensitive to erlotinib, becoming resistant. As a result, erlotinib cannot be an effective long-term treatment unless combined with ErbB3 antagonists^13^. Therefore, it is believed that inhibition of ErbB3 signaling may be required to overcome therapeutic resistance and effectively treat cancers.

*Radix Paeoniae Alba* has been frequently used as an important ingredient in many traditional prescriptions and is commonly used for treating digestive system diseases. In our clinical treatment, *Radix Paeoniae Alba* together with other herbs showed significant anti-cancer efficacy among patients with pancreatic cancer^23^, and *in vitro* experiments showed that the crude water extract of *Radix Paeoniae Alba* inhibited ErbB3 phosphorylation and retarded PI3K/Akt signaling in the pancreatic cancer cell lines BxPC-3 and L3.6pl (Figure S1). Thus, it is reasonable to speculate that PF, the principal bioactive component of *Radix Paeoniae Alba*^24^, could enhance the efficacy of erlotinib by interfering with the ErbB3/PI3K/Akt signaling pathway. Therefore, in this study, we investigated the capacity of PF to sensitize erlotinib-induced inhibition of cell viability in pancreatic cancer cell lines.

## Results

### Effects of Erlotinib and PF on the Viability of BxPC-3 and L3.6pl cells

**Figure 1:**
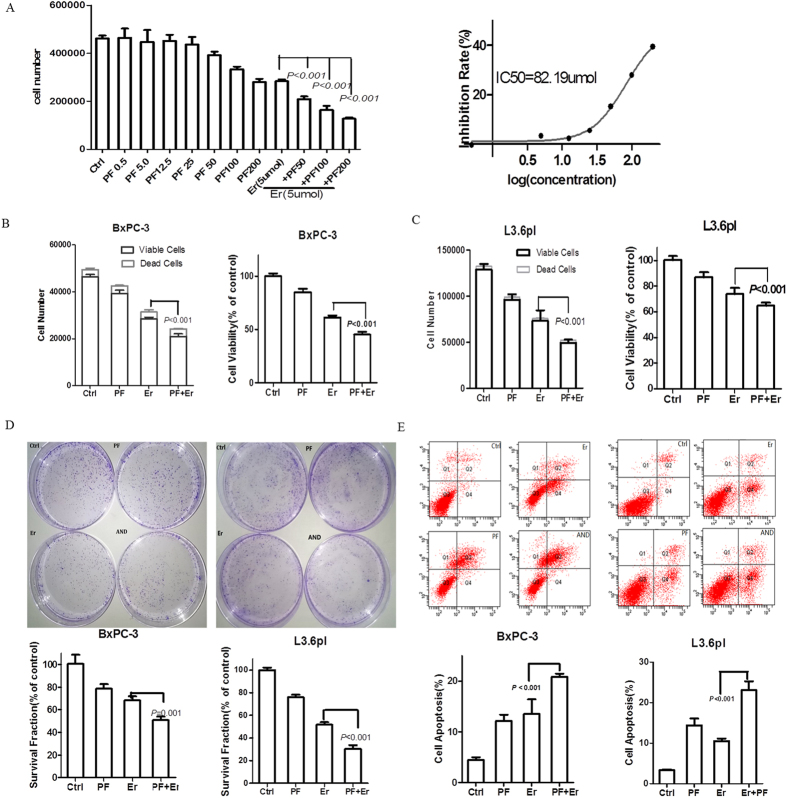
Effects of PF and Erlotinib on High ErbB3-Expressing Pancreatic Cancer Cells. (**A**) BxPC-3 cells were exposed to serial concentrations of PF for 48 h. The half maximal (50%) inhibitory concentration (IC_50_) value for PF in the BxPC-3 cell line is shown as a reference. The inhibitory effect of the combination of PF (50 μmol/L) and erlotinib (5 μmol/L) in BxPC-3 (**B**) and L3.6pl cell lines (**C**) using Trypan Blue Exclusion (Left) and MTS assays (Right). There was a significant reduction in the colony formation in BxPC-3 and L3.6pl cells treated with the combination compared to cells treated with either drug alone (**D**). Apoptotic effects of PF (50 μmol/L), erlotinib (5 μmol/L) and a combination of PF (50 μmol/L) and erlotinib (5 μmol/L) using Annexin-V/PI method. There was a significant potentiation of apoptosis in BxPC-3 cells and L3.6pl cells treated with the combination compared to cells treated with either drug alone (**E**).

As the first step of our investigation, we assessed the effect of PF on the viability of high ErbB3-expressing human pancreatic cancer BxPC-3 and L3.6pl cells. Cells were treated with increasing concentrations of PF for 48 h and Trypan Blue Exclusion analysis was used to assess the effect of these treatments on cell viability, principally to determine the proportion of live and dead cells within the population. As shown in Fig. 1A, PF treatment resulted in a significant concentration-dependent decrease in the number of viable cells starting at a concentration of 50 μmol/L with an inhibition rate of 15.24% (*P* < 0.001). The half maximal (50%) inhibitory concentration (IC_50_) value for PF was 82.19 μmol/L in the BxPC-3 cell line. Different concentrations of erlotinib (2 μmol/L, 5 μmol/L, 10 μmol/L and 20 μmol/L) were examined in BxPC-3cell line, and the inhibition rates were found to be 20.18%, 38.73%, 53.44%, 65.50%, respectively. We further examined the inhibitory effect of the combination of PF (50 μmol/L) and erlotinib (5 μmol/L) on high ErbB3-expressing pancreatic cancer cell lines BxPC-3(Fig. 1B) and L3.6pl (Fig. 1C) using Trypan Blue Exclusion and MTS assays. Co-treatment with PF enhanced the ability of erlotinib to induce growth inhibition in BxPC-3 and L3.6pl cells.

Next, we determined the effect of PF on the ability of cells to form colonies. Similarly, the combination of PF and erlotinib significantly reduced the colony number in both BxPC-3 and L3.6pl cell lines, as shown by the colony formation assay (Fig. 1D). The underlying mechanism of cell viability inhibition was further studied by determining the apoptotic effects of PF (50 μmol/L), erlotinib (5 μmol/L) or a combination of PF (50 μmol/L) and erlotinib (5 μmol/L) using the Annexin-V/PI method. There was a significant increase of apoptotic cells (D2 + D4) treated by the combination of PF and erlotinib compared to either agent alone in both high ErbB3-expressing BxPC-3 and L3.6pl cell lines (Fig. 1E). Consistent with the cell viability assays, these results again indicated that PF enhanced the efficacy of erlotinib in the high ErbB3-expressing cell lines BxPC-3 and L3.6pl.

### Effects of Erlotinib and PF on ErbB3-deficient MIAPaCa-2 cells

**Figure 2:**
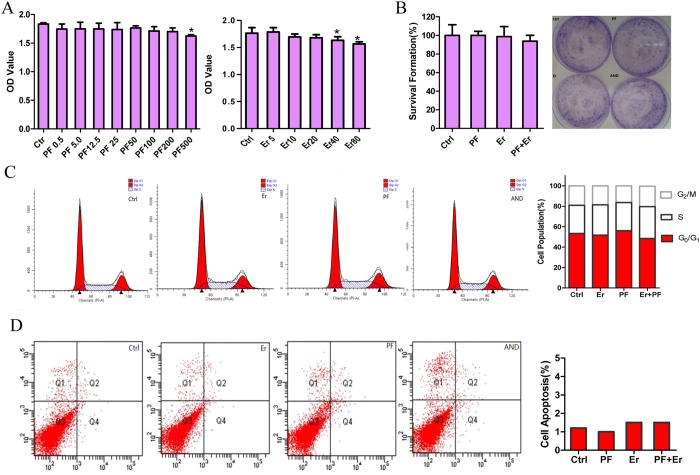
Both PF and erlotinb treatment did not influence the growth of ErbB3-deficient pancreatic cancer cells. When exposed to different concentrations of PF and erlotinb, the proliferation of MIAPaCa-2 cells was not influenced (**A**). Similar treatments of PF (50 μmol/L), erlotinib (5 μmol/L) and the combination did not influence colony formation (**B**), cell apoptosis (**C**) and cell cycle (**D**) in ErbB3-deficient MIAPaCa-2 cells.

As a proof concept, we aimed to detect the influence of PF on MIAPaCa-2 cells which lack detectable ErbB3 and exhibit pronounced erlotinib resistance. In our research, when exposed to similar PF concentrations, the proliferation of MIAPaCa-2 cells was not influenced. MIAPaCa-2 proliferation did not change following erlotinib treatments at the concentrations of 2 μmol/L, 5 μmol/L, 10 μmol/L and 20 μmol/L as tested by MTS assay (Fig. 2A). At 40 μmol/L and 80 μmol/L, cell proliferation was inhibited by erlotinib. However, growth inhibitory effects observed as a result of concentrations of erlotinib up to 10 μmol/L are not biologically and clinically significant as the average steady-state concentration of erlotinib is 11.0 μmol/L^25^. Similar treatments with PF (50 μmol/L), erlotinib (5 μmol/L) and the combination did not influence in colony formation (Fig. 2B), cell apoptosis (Fig. 2C) and cell cycle (Fig. 2D) in ErbB3-deficient MIAPaCa-2 cells.

### ErbB3/PI3K/Akt pathway involved in the inhibitory effect of PF on cell viability

**Figure 3:**
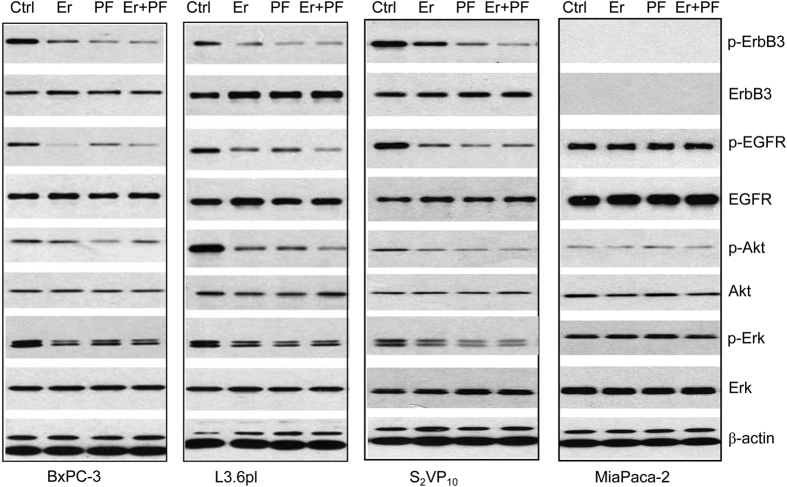
Effect of Erlotinib, PF, and Combination Treatment on the Protein Expression in Pancreatic Cancer Cells. In high ErbB3-expressing BxPC-3, L3.6pl and S_2_VP_10_ cell lines, ErbB3 phosphorylation was reduced to a negligible level following the treatment with PF. Dually targeting ErbB3 and EGFR through the combination of PF and erlotinib displayed dramatic inhibition of the activitions of both EGFR/MAPK/Erk and ErbB3/PI3K/Akt pathways.

To clarify the mechanism by which PF enhanced the anti-tumor activities of erlotinib, the activitions of ErbB3, EGFR and their downstream molecules were examined. Consistent with previous reports^12^,^13^,^14^, ErbB3 is overexpressed in BxPC-3, L3.6pl and S_2_VP_10_ cell lines, whereas it was barely detectable in MiaPace-2 cells. As shown by the western blot analysis (Fig. 3), neither PF nor erlotinib affected the expression of total ErbB3 protein in the pancreatic cancer cell lines. Most strikingly, ErbB3 phosphorylation was reduced to a negligible level in high ErbB3-expressing BxPC-3, L3.6pl and S_2_VP_10_ cell lines following the treatment with PF. The inhibitory effect of a single treatment of erlotinib on ErbB3 and Akt activitions was modest as well as the single treatment of PF on EGFR and Erk activitions, whereas the combination of PF and erlotinib substantially suppressed both EGFR/MAPK/Erk and ErbB3/PI3K/Akt activitions in ErbB3-expressing BxPC-3, L3.6pl and S_2_VP_10_ cell lines. Different cell lines showed variable levels of ErbB-3/Akt phosphorylation and EGFR/Erk phosphorylation after the treatment with combination of PF and erlotinib. In L3.6pl and S_2_VP_10_ cell lines, there was a more significant reduction in activation of ErbB3/Akt than in activation of EGFR/MAPK/Erk, while in BxPC-3, the reduction of phosphorylation of EGFR was more dramatic than reduction of phosphorylation of Akt. This may be related to the type of cells.

The inhibitory effect of single PF, erlotinib or the combination on EGFR/MAPK/Erk and ErbB3/PI3K/Akt activitions were not shown in MIAPaCa-2, which was to be expected since MIAPaCa-2 was the only cell line with undectable levels of ErbB3 protein expression.

### Effects of PF and Erlotinib on Xenografts Tumor Models

To further evaluate the anti-tumor efficacy of the combination of PF and erlotinib, BALB/C nude mice bearing BxPC-3 xenografts were treated orally with PF and erlotinib. After 40 days of treatment, the numbers of mice in the control, PF, erlotinib and combination group were 8, 8, 6, and 7, respectively. The two deaths in the erlotinib group were due to serious rashes and diarrhea after 21 days of treatment. The cause of death in the combination group was unknown.

**Figure 4:**
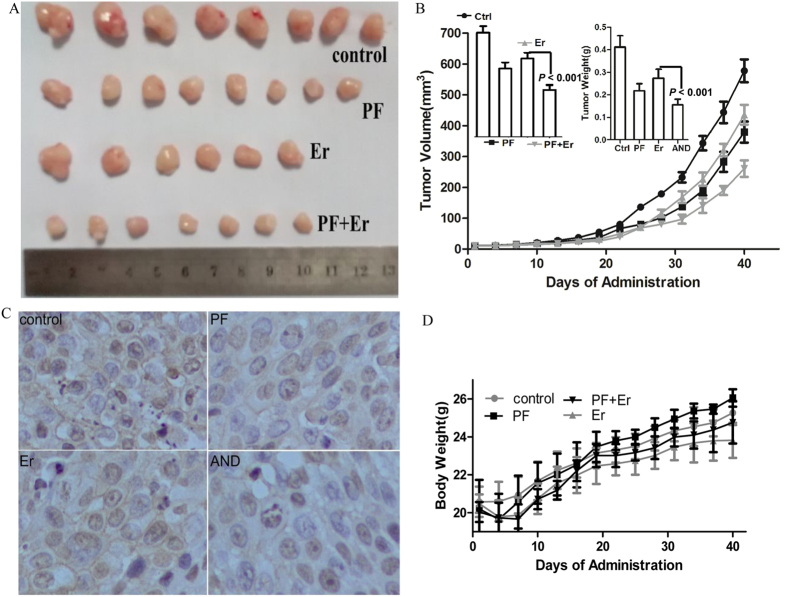
PF Augmented *in Vivo* Therapeutic Effects of Erlotinib on the BxPC-3 Transplanted Tumors. (**A**) Representative image of excised tumors (**B**) Effects of erlotinib, PF or Combination treatment on tumor volume and weight. The volume curve shows the change in tumor volume, and the left histogram shows the final tumor volume. On the 40th day, the implanted tumors were excised and weighted (right histogram). (**C**) Expression of phosphorylated ErbB3 in tumor tissues. Left, images are shown with phosphorylated ErbB3 positive areas (400×). (**D**) Effect of erlotinib, PF or combination on mouse weight.

Treatment with erlotinib or PF alone induced significant growth delay of the BxPC-3 xenografted tumor compared to untreated controls, as shown in the Fig. 4A,B. The tumor weight was reduced by 46.0% and 32.15% in the PF-treated and erlotinib-treated groups, respectively. The combination drastically and effectively suppressed tumor growth compared to either drug alone with the tumor weight being reduced by 61.46% (*P *< 0.001 for both). Next, phosphorylation of ErbB3 in tumor xenografts was assessed. Consistent with the *in vitro* observations, p-ErbB3 was reduced by PF treatment. Furthermore, the combination of PF and erlotinib led to a more drastic reduction of p-ErbB3 compared to either drug alone (Fig. 4C).

The increase in body weight was 4.69 g for the control group, 5.85 g for the PF group, 3.33 g for the erlotinib group and 4.71 g for the combination group. No significant differences in body weight change were observed in the combination groups compared to the control group (*P *> 0.05, Fig. 4D). PF treatment did not lead to toxicity in mice. As shown in Table 1, the organ indexes for the heart, liver, and kidney were in generally similar between the control and each of the treatment groups (*P *> 0.05 for all).

## Discussion

*Radix Paeoniae Alba* has been frequently used since the period of the Han Dynasty (second century) as an anti-inflammatory, hepatoprotective and neuroprotective agent^26^,^27^,^28^,^29^,^30^,^31^. PF, the principal component of Total glucosides of paeonia (TGP), has also been shown to exert anti-cancer and anti-proliferative activity in cultured cells as well as in animal models. Previous studies have shown that PF induces cancer cell apoptosis in gastric cancer cells, liver cancer cells, ovarian cancer cells and leukemia cells through various mechanisms, such as the modulation of the NF-kB activation pathway, Bcl-2 and Bax expression, or the MDM2-p53 pathway^32^,^33^,^34^,^35^,^36^,^37^,^38^. In these published reports, the PF concentrations were prescribed at a milligram level, which limits its use in the clinical setting. In our clinical treatment, *Radix Paeoniae Alba* together with other herbs showed significant anticancer efficacy among patients with pancreatic cancer, and *in vitro* experiments showed that the crude water extract of *Radix Paeoniae Alba* inhibited ErbB3 phosphorylation and retarded PI3K/Akt signaling in pancreatic cancer cells. In this study, we illustrated that PF at low concentrations (50 μmol/L) showed inhibitory effects on high ErbB3-expressing pancreatic cancer cells by inducing apoptosis. More importantly, for the first time, we found that the anti-tumor mechanism of PF was highly associated with the inhibition of ErbB3 phosphorylation.

Over-expressed ErbB3 protein is the preferred dimerization partner of EGFR and is involved in the erlotinib response in pancreatic cancer cells^12^,^13^,^14^, but it could also influence pancreatic cancer tumorigenesis, as high expression of ErbB3 correlates with advanced disease stage and decreased overall survival^39^,^40^. Although ErbB3 has a very weak intracellular tyrosine kinase activity, its transactivation by other members of the EGFR family induces direct phosphorylation of the six binding sites for the p85 regulatory subunit of PI3K, resulting in activation of the Akt signaling cascade^8^. By inhibiting transphosphorylation of ErbB3 via EGFR, erlotinib could interfere with PI3K/Akt signaling in some degree^14^,^16^. However, the inhibition of ErbB3 phosphorylation by erlotinib is merely transient, which results in the reactivation of ErbB3/PI3K/Akt signaling^8^,^14^,^16^,^17^. Moreover, ErbB3 is also activated by other receptor tyrosine kinases (notably MET, IGF-1R, or TRK-B), which also leads to patients who initially responded to erlotinib ultimately became refractory to treatment^19^,^20^,^21^,^22^. Therefore, ErbB3/PI3K/Akt signaling plays an important role in erlotinib treatment. Nonetheless, combinatorial therapies including ErbB3 and EGFR targeting may increase the effectiveness of targeted therapies.

The approach for combining erlotinib and PF in our study was based on the molecular detected by western blot. Although the inhibitory effects of single erlotinib treatment on ErbB3/PI3K/Akt activity or PF treatment alone on EGFR/MAPK/Erk activities were modest, dually targeting ErbB3 and EGFR through the combination of PF and erlotinib displayed dramatic inhibition of both EGFR and ErbB3 activity. This combination allowed erlotinib to efficiently inhibit the proliferation of pancreatic cancer cells. These results were also recapitulated *in vivo* using mouse xenograft models and immunohistochemical staining, strengthening our underlying rationale for this novel treatment method for patients with pancreatic cancer. Conversely, cells lacking the ErbB3 molecular signature, such as MIAPaCa-2 cells, would not benefit from either PF alone or the combination of PF and erlotinib. Previous studies have shown that erlotinib inhibits the proliferation of all the ErbB3-expressing cell lines but does not affect proliferation of ErbB3-deficient MIAPaCa-2 cells, which displayed persistent PI3K/Akt activation after erlotinib treatment^13^. Activation of PI3K/Akt in MIAPaCa-2 cells may therefore rely on alternative signaling cascades. In our study, PF did not inhibit proliferation of MIAPaCa-2 cells but showed sensitive inhibitory effects on the proliferation and ErbB3/PI3K/Akt activation of ErbB3-expressing cell lines. This suggests that PF does not influence the PI3K/Akt activation directly; its function relied on the function of ErbB3.

Another principle behind combination PF and erlotinib treatment is that the side effects of two drugs should not overlap. The most effective strategy to specifically target ErbB3 is the use of monoclonal antibodies. By combining monoclonal antibodies with TKIs, the previously suggested combination of cetuximab with BIBW-2992 (an irreversible EGFR/HER2-TKI) raised concerns about toxicity because these drugs share common side effects, such as diarrhea and skin eruption^15^. Therefore, the clinical feasibility remains unclear in terms of tolerance. In contrast, PF has been widely used for long periods of time with negligible toxicity. Rather, PF has shown an anti-inflammatory and immunoregulation effects^41^, and it may therefore lessen the skin problems caused by erlotinib.

In summary, PF enhanced the effect of erlotinib in ErbB3-expressing cell lines by directly suppressing ErbB3 activation. Our results support the hypothesis that combination treatment with PF and erlotinib is a promising strategy to enhance proliferation inhibition in ErbB3-expressing pancreatic cancer cells. Moreover, the choice of PF as a co-treatment in pancreatic cancer and its application in the clinic was also favorable based on its minimal toxicity.

## Materials and Methods

### Drugs and Reagents

**Figure 5:**
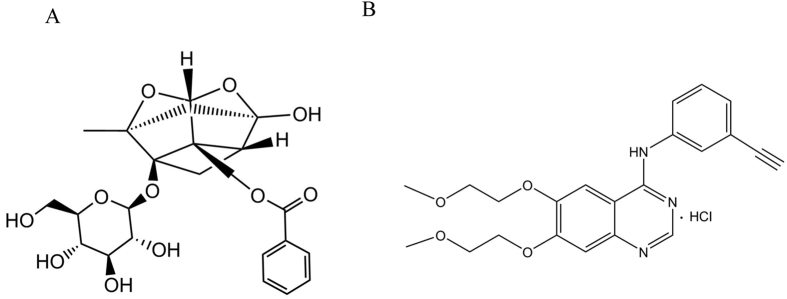
Chemical structures of PF (**A**) and erlotinib (**B**).

Erlotinib (Tarceva, OSI-774) was purchased from OSI Pharmaceuticals (Selleck, USA). PF (purity greater than 99%) was purchased from the HuanYu Biotechnology Development Co., Ltd Products (Beijing, China). The molecular formulas of the two drugs were shown in Fig. 5A,B. PF was dissolved in DMEM or RPMI 1640 medium (Hyclone), and then diluted as needed in cell culture medium. Erlotinib was dissolved in DMSO at a concentration of 10 mmol. For experiments, the final concentrations of the test compounds were prepared by diluting the stock solution with DMEM or RPMI 1640. Control cultures received the carrier solvent (0.1% DMSO).

### Cells Culture

Human pancreatic cancer cell lines were chosen for this study based on their constitutive levels of ErbB3 expression. BxPC-3, L3.6pl and S_2_VP_10_ were reported as high ErbB3-expressing cell lines, and MIAPaCa-2 was an ErbB3-deficient cell line^12^,^13^,^14^. BxPC-3 and MIAPaCa-2 were obtained from Tianjin Medical University Cancer Research Institute. L3.6pl and S_2_VP_10_ cells were obtained from Department of Medicine, University of Alabama at Birmingham. BxPC-3 cells were maintained in RPMI 1640 supplemented with 10% fetal bovine serum (Hyclone). L3.6pl, S_2_VP_10_ and MIAPaCa-2 cells were grown in DMEM supplemented with 10% fetal bovine serum. All cell lines were cultured at 37 °C in a humidified atmosphere of 95% air and 5% CO_2_.

### Cell Proliferation Assay

Exponentially growing pancreatic cancer cells (1 × 10^4^) were plated in 24-well plates. Upon attachment overnight, cells were treated with PF, erlotinib or a combination of both compounds for 48 h. Cells were collected following trypsinization and counted using 0.4% Trypan Blue to verify the viability. Each treatment was performed in four replicate wells and repeated in at least three independent experimental trials. IC_50_ (50% cell growth inhibition) value of PF on BxPC-3 cells was calculated graphically. Cell viability was also evaluated using the MTS assay. The cells were seeded in 96-well culture plates at a concentration of 2 × 10^3^ cells/well. Cell viability was then assessed by adding 20 μL of 10 mg/mL 3-[4,5-dimethylthiazol-2-yl]-2,5-(3-carboxymethoxyphenyl)-2-(4-sulfophenyl)-2H-tetrazolium, inner salt (MTS, Sigma) to 80 μL of culture medium after 48 h. The optical density was measured at 490 nm using a Multiskan EX (Thermo, Finland).

### Clonogenic Assay

Survival of cells treated with erlotinib, PF, or the combination was also defined as the ability to maintain their clonogenic capacity and to form colonies. Briefly, BxPC-3, L3.6pl and MIAPaCa-2 cells were trypsinized, counted, and seeded for colony formation in 60-mm dishes at range of 100 to 1000 cells/dish. Following exposure to erlotinib, PF, or the combination for 10 to 14 days, colonies were stained with crystal violet and manually counted. Colonies consisting of 50 cells or more were scored, and four replicate dishes were counted for each treatment.

### Apoptosis analysis by flow cytometry

Cellular apoptosis was examined by using an Annexin V–FITC apoptosis detection kit (BD, New York, USA). BxPC-3, L3.6pl and MIAPaCa-2 cells were seeded at a density of 2 × 10^5^ cells into a 6-well plate. After being treated with PF, erlotinib and the combination, the cells were incubated for 48 h. The cells were then harvested with trypsin and washed in PBS. After centrifugation at 1000 rpm, the supernatant was removed and then the cells were suspended in a stain containing AnnexinV‐FITC and propidium iodide (PI). The stained cells were incubated at room temperature for 15 min in the dark. The cells were then analyzed using FACS Calibur flow cytometry (BD, New York, USA). Each treatment was performed in replicate.

### Protein Extraction and Western Blot Analysis

Exponentially growing BxPC-3, L3.6pl, S_2_VP_10_ and MIAPaCa-2 cells were harvested by trypsinization to evaluate the effects of treatment on ErbB3 (dilution 1: 800, Santa Cruz Biotechnology), ErbB3-pTyr1197 (dilution 1: 800, Santa Cruz Biotechnology), EGFR, EGFR-pTyr1068, Akt, Akt-pS473, Erk, Erk-pT202/Y204, and β-actin (dilution 1: 1000, Abcam) expression. After centrifugation, the cell pellet was lysed by lysis buffer containing 25 mM Tris-HCl, pH 7.5, 150 mM NaCl, 1% TritonX100 v/v, 1 mM EDTA, 1 mM EGTA, 1 mM Na_3_VO_4_, 1 mM DTT, 1 mM PMSF, and 1 μg/ml of protease inhibitor cocktail. Equal amounts of cell lysate were subjected to 7% to 12% SDS-PAGE for separation and electrophoretically transferred to a nitrocellulose membrane. After transfer to a membrane, the protein blots were incubated in the presence of primary antibody at 4 °C overnight. After incubation with horseradish peroxidaseconjugated secondary antibody, the signals of detected protein were visualized by using an ECL reaction.

### *In vivo* analysis of Combined Drug Effects

All experiments were approved by the Tianjin Cancer Institutional Animal ethics Committee and adhered to the guidelines of National Institutes of Health Guidelines for Animal Care. Animals were maintained under standard laboratory conditions and all procedures were performed in conformity with Tianjin Cancer Institutional Animal ethics Committee (protocol #2014055). Male BALB/c nude mice (4 weeks, weighing 18–20 g) were purchased from the Vital Revier Experimental Animal Technical Company (Beijing). The nude mice were injected subcutaneously with BxPC-3 pancreatic cancer cells (0.1 ml, 1 × 10^7^ cells). To guarantee the homogeneous tumor size, we transplanted subcutaneous tumors to 32 other male BALB/c nude mice. Briefly, after 2 weeks, a solitary tumor had grown at the injection site in each mouse. Necrotic and non-cancerous tissues were removed and then the tumor was divided into approximately 1-mm^3^ fragments. A fragment of BxPC-3 tumor was subcutaneously implanted using an implanting needle (Natsume, Tokyo, Japan) into the subcutaneous tissue of left abdomen. The mice were allocated into four groups (n = 8) according to treatment: 1) normal saline (control group); 2) PF 500 mg/kg; 3) erlotinib 50 mg/kg; and 4) erlotinib combined with PF. Drug administration began on the next day of inoculation, and each animal received the treatment drug once a day for 40 days via intragastric injection administration. Tumors were measured individually every three days in two dimensions (length and width) using calipers. The tumor volume (mm^3^) was calculated as V = length × width^2^/2. On the 40th day, the animals were weighed and sacrificed. The implanted tumors were excised and weighed.

### Immunohistochemical analysis

Tumor samples were fixed in 10% buffered formalin. Paraffin embedded, 5 mm thin sections were deparaffinized and stained with primary antibody against ErbB3-pTyr1197 (dilution 1: 300) overnight at 4 °C. The sections were biotinylated secondary antibody for 30 min at room temperature. Thereafter, sections were incubated with 3,3-diaminobenzidine (Dako) working solution, and counter-stained with hematoxylin. To quantify ErbB3-pTyr1197 expression, the sections were photographed with a digital camera at 400× magnification.

### Imaging and Statistics

Images were processed using Photoshop CS4 (Adobe) and any adjustments were made to the entire image and equally for each genotype. The student’s *t* test was used to evaluate the significance of differences between two samples, and ANOVA was used to evaluate differences among three or more groups. Differences between samples were considered statistically significant when *P* < 0.05. Data were analyzed using SPSS19.0 and GraphPad Prism 4 software (Version 4.03).

## Additional Information

**How to cite this article**: Hao, J. *et al*. Paeoniflorin Potentiates the Inhibitory Effects of Erlotinib in Pancreatic Cancer Cell Lines by Reducing ErbB3 Phosphorylation. *Sci. Rep.* **6**, 32809; doi: 10.1038/srep32809 (2016).

## Supplementary Material

Supplementary Information

## Figures and Tables

**Table 1:** Effect of PF and Er on Organ Index (mg/g, ± s).

Group	n	Organ Index			
		Heart	Liver	Kidney	Spleen
Control	8	5.919 ± 0.791	61.393 ± 8.497	18.823 ± 1.721	8.634 ± 1.952
PF	8	5.738 ± 0.970	56.889 ± 2.466	17.664 ± 0.640	7.979 ± 0.916
Er	6	6.492 ± 0.995	59.263 ± 3.218	18.916 ± 0.996	8.584 ± 1.422
PF + Er	7	6.768 ± 0.199	54.457 ± 2.263	18.045 ± 1.121	8.671 ± 0.628
