# Supplementary material for: Paeoniflorin Potentiates the Inhibitory Effects of Erlotinib in Pancreatic Cancer Cell Lines by Reducing ErbB3 Phosphorylation
Source: Sci Rep. 2016 Sep 9;6:32809. doi: 10.1038/srep32809 (PMC5016851; doi:10.1038/srep32809)

Paeoniflorin Potentiates the Inhibitory Effects of Erlotinib in Pancreatic Cancer Cell Lines by Reducing ErbB3 Phosphorylation

Jian Hao^1#^, Xue Yang^1#^, Xiu-li Ding^1^, Lei-ming Guo^2^, Cui-hong Zhu^1^, Wei Ji^3^, Tong Zhou^2^, Xiong-zhi Wu^1^*

^#^These authors contributed equally to this study and share first authorship.

^1^Zhong-Shan-Men Inpatient Department；National Clinical Research Center for Cancer; Key Laboratory of Cancer Prevention and Therapy; Tianjin Medical University Cancer Institute and Hospital Tianjin, 300060, China.

Jian Hao, haojian1111520@126.com; Xue Yang, yang.xue.cool@163.com; Xiu-li Ding, dxl_123@outlook.com；Cui-hong Zhu，zch890127@163.com

^2^Clinical Immunology and Rheumatology, Medicine Department of University of Alabama at Birmingham, Birmingham, Alabama, USA.

Lei-ming Guo, ray2007_guo@hotmail.com; Tong Zhou, tzhou@uab.edu

^3^Opening Cancer Laboratory, Tianjin Medical University Cancer Institute and Hospital, Tianjin 300060, China. Ji Wei, jiwei217@126.com

*Correspondence to: Huan-Hu-Xi Road, Ti-Yuan-Bei, He-Xi District, Zhong-Shan-Men Inpatient Department, Tianjin Medical University Cancer Institute and Hospital, Tianjin, 300060, China Telephone: +86-22-23921723 Fax: +86-22-23921723. E-mail: wuxiongzhi@163.com

Figure S-1

The effects of different herb crude water extract on ErbB3 phosphorylation and retarded PI3K/Akt signaling in the pancreatic cancer cell lines BxPC-3 and L3.6pl. PA: Paeoniae Alba


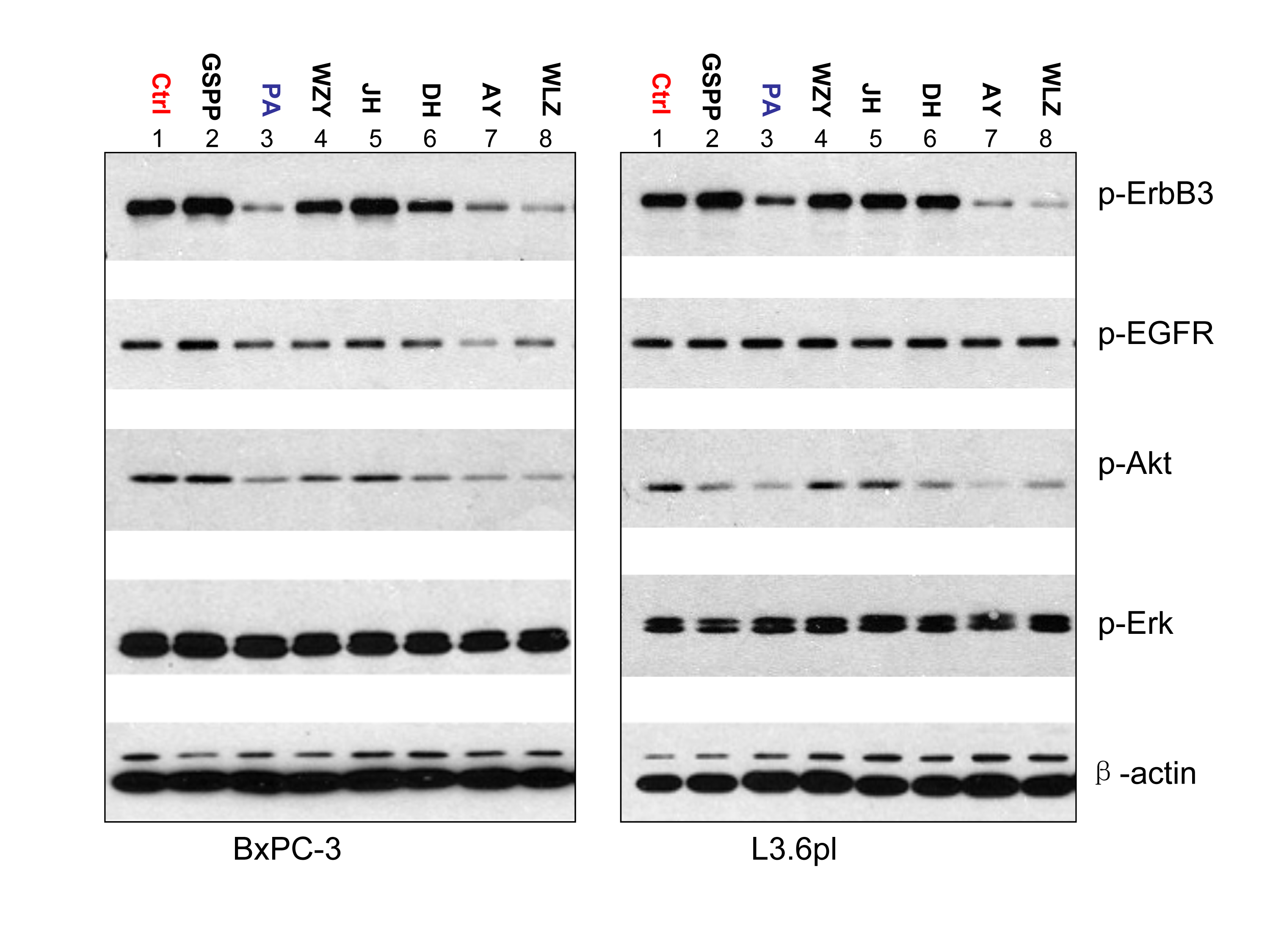

Supplement: Supplementary Information [file srep32809-s1.doc]
